# High T3 Induces *β*-Cell Insulin Resistance via Endoplasmic Reticulum Stress

**DOI:** 10.1155/2020/5287108

**Published:** 2020-07-22

**Authors:** Bo Liang, Liyun Liu, Huibin Huang, Liangyi Li, Jingxiong Zhou

**Affiliations:** ^1^Department of Endocrinology, The Second Affiliated Hospital of Fujian Medical University, Quanzhou 362000, China; ^2^Department of Pediatrics, The First Hospital of Quanzhou Affiliated to Fujian Medical University, Quanzhou 350005, China

## Abstract

Hyperthyroidism can cause glucose metabolism disorders and insulin resistance. Insulin resistance in muscle and adipose tissues has been extensively studied, whereas investigations on *β*-cell insulin resistance are limited. This study preliminarily explored the effects of high T3 levels on *β*-cell line (MIN6) insulin resistance, as well as the roles of endoplasmic reticulum stress (ERS). In this study, we treated *β*-cell line with T3, with or without an inhibitor of phosphotyrosine phosphatases (PTPs, sodium vanadate) or ERS inhibitor (4-PBA). The results indicated that high levels of T3 significantly inhibited insulin secretion in *β*-cell line. In addition, we observed an upregulation of p-IRS-1^ser307^ and downregulation of Akt. These results can be corrected by sodium vanadate. Moreover, high T3 levels upregulate the ERS-related proteins PERK, IRE1, ATF6, and GRP78, as well as ERS-related apoptosis CHOP and caspase-12. Similarly, this change can be corrected by 4-PBA. These results suggest that high T3 levels can induce insulin resistance in *β*-cell line by activating ERS and the apoptotic pathway.

## 1. Introduction

About 70% to 75% of hyperthyroidism cases are associated with glucose metabolism disorders and insulin resistance [1, 2]. The effect of thyroid hormones on glucose metabolism is mainly through T3, which binds to T3 receptors in *β*-cell nuclei and regulates insulin signal transduction as well as secretion [3, 4]. However, the mechanism of high T3 levels on insulin resistance in *β*-cells has not been elucidated.

The endoplasmic reticulum is an organelle in eukaryotic cells, except for mammalian red blood cells, that is mainly responsible for the synthesis, processing, and secretion of proteins, as well as the metabolism of lipids and sugars [5]. A variety of adverse factors can block the synthesis and processing of proteins, such as chemical toxicants, high glucose, low temperature, and hypoxia [6]. These factors can cause unfolded or misfolded proteins to accumulate in the ER cavity and induce endoplasmic reticulum stress (ERS) [7, 8]. A growing body of evidence suggests that insulin resistance in *β*-cells induced by ERS affects insulin signal transduction [9, 10]. All of these pathways can inhibit insulin signal transduction and cause insulin resistance [11, 12]. In addition, ERS induced the upregulation of CHOP and caspase-12, which cause *β*-cell apoptosis in diabetes [13]. The purpose of this study was to investigate the effects of high T3 levels on insulin signal transduction, insulin secretion in *β*-cells, and the role of ERS in this process.

## 2. Materials and Methods

### 2.1. Materials

The MIN6 cell line was obtained from the Institute of Basic Medicine, Chinese Academy of Medical Sciences (Beijing, China). Liothyronine sodium (T3 or liothyronine) was purchased from Suzhou Famu Biomedical Technology Co., Ltd. (Suzhou, China), sodium orthovanadate (an inhibitor of phosphotyrosine phosphatases) was purchased from Shanghai Macklin Biochemical Co., Ltd. (Shanghai, China), and phenylbutyric acid (4-PBA) was obtained from Sigma (USA). Western blot reagents were obtained from Cell Signaling Technology (CST, USA), and cell culture materials were obtained from Thermos.

### 2.2. Cell Culture

MIN6 cells were cultured in DMEM with 10% fetal bovine serum in a 37°C incubator with 5% CO_2_. The culture medium was replaced every two days, and the cells were routinely passaged every seven days. Cells were harvested for assays when cell confluency reached 100%.

### 2.3. CCK8 Assay

In this study, different concentrations of T3 were used to stimulate MIN6 cells, and then, the CCK8 method was used to detect the effect of T3 on the activity of MIN6 cells.

### 2.4. Cell Treatment with Liothyronine Sodium

When the cells reached 100% confluency, liothyronine sodium was added into the culture medium at a concentration of 5 pg/mL or 20 pg/mL (final concentration) and was designated as normal- or high-concentration FT3 treatment group (N or H group). All of the treatments were continuously cultured for 48 h.

### 2.5. Administration of Sodium Orthovanadate and 4-PBA

Sodium orthovanadate (final concentration: 5 *μ*mol/L) and ERS inhibitor 4-PBA (final concentration 1 mmol/L) were independently applied to the H group for 48 h [14].

### 2.6. Western Blot Analysis

Protein samples were prepared as follows: cell pellets were lysed with cold RIPA cleavage buffer and centrifuged at 12,000 g for 20 min at 4°C, and protein concentrations were determined using the BCA method. After electrophoresis with 10% polyacrylamide gel, the proteins were transferred onto a PVDF membrane using standard semidry transblot procedures. After blocking with 5% BSA in PBS for 2 h at room temperature, the membrane was incubated with a primary antibody overnight at 4°C. After three washes with TBST buffer, the membrane was incubated with HRP-conjugated goat anti-rabbit IgG or goat anti-mouse IgG for 1 h at room temperature. After extra washes with TBST buffer, the membrane was developed using the standard ECL method.

### 2.7. Enzyme-Linked Immunosorbent Assay

The culture media of the cell plates were transferred into EP tubes. The supernatants were centrifuged at 1,000 rpm for 5 min and then placed into an EP tube. Insulin concentrations were measured using the mouse insulin enzyme-linked immunosorbent assay (ELISA) kit (ExCell Bio. Co., Ltd., Shanghai, China).

### 2.8. Statistical Analysis

SPSS 21.0 software was employed for statistical analysis. Statistical analysis was performed using Student's *t*-test when comparing two groups or ANOVA with Bonferroni post hoc test when comparing three or more groups. Differences with *p* <0.05 were considered to be statistically significant.

## 3. Results

### 3.1. The Effect of Different Concentrations of T3 on the Activity of MIN6 Cells

**Figure 1:**
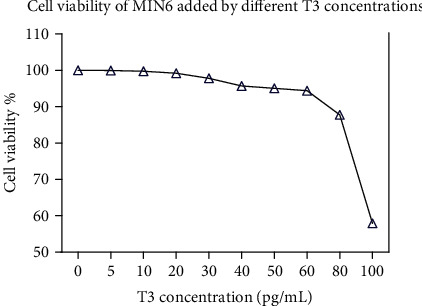
The effect of different concentrations of T3 on the activity of MIN6 cells. In this study, the CCK8 method was used to detect the effect of different concentrations of T3 on the activity of MIN6 cells. When the concentration of T3 is lower than 80 pg/mL, there is no effect on the activity of MIN6 cells.

The results of the CCK8 test showed that when the concentration of T3 was lower than 80 pg/mL, the cell activity was not significantly affected (Figure 1).

### 3.2. High T3 Induces Insulin Resistance in Pancreatic Cells

**Figure 2:**
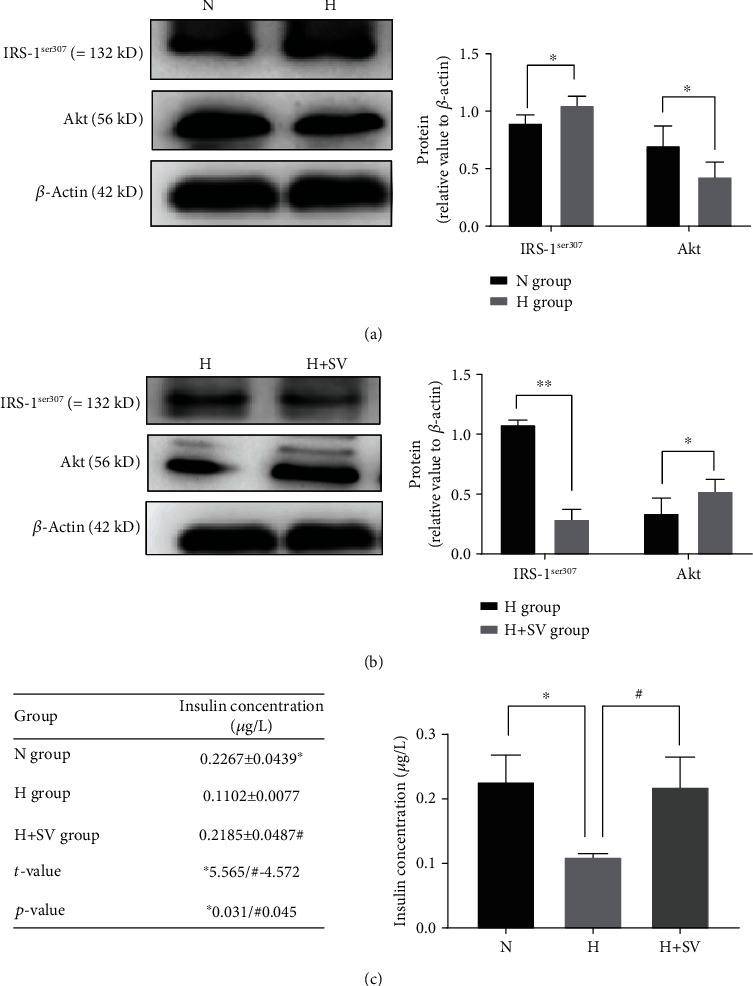
Expression of p-IRS-1^ser307^, Akt, and insulin in *β*-cells treated with different concentrations of T3. N group: normal-concentration T3 (5 pg/mL); H group: high-concentration T3 (20 pg/mL); H+SV group: high-concentration T3 (20 pg/mL)+sodium orthovanadate (5 *μ*mol/L). (a) Western blotting analysis showed that the expression of p-IRS-1^ser307^ in the H group was significantly higher than that in the N group, whereas Akt was lower. (b) p-IRS-1^ser307^ in the H+SV group was significantly lower than that in the H group, whereas Akt was higher than that in the H group. (c) ELISA showed that the concentration of insulin in the supernatant of the N group was significantly higher than that of the H group, whereas that of the H group was significantly lower than that of the H+SV group (*n* = 5, group = 3, ^∗^*p* < 0.05, ^∗∗^*p* < 0.01). ELISA: enzyme-linked immunosorbent assay.

Different concentrations of T3 were used to treat islet *β*-cells. Then, we collected the total proteins of these cells. Western blotting analysis showed that compared with the normal-concentration T3 group, the high-concentration T3 group exhibited an upregulation of IRS-1^ser307^ and downregulation of Akt (Figure 2(a)). Then, we used ELISA to detect the insulin content in the MIN6. ELISA results showed that high concentrations of T3 decrease insulin secretion in islet *β*-cells. Those processes were reversed by sodium orthovanadate by inhibiting insulin resistance (Figures 2(b) and 2(c)).

### 3.3. High T3 Induces Insulin Resistance via ERS in Pancreatic Cells

**Figure 3:**
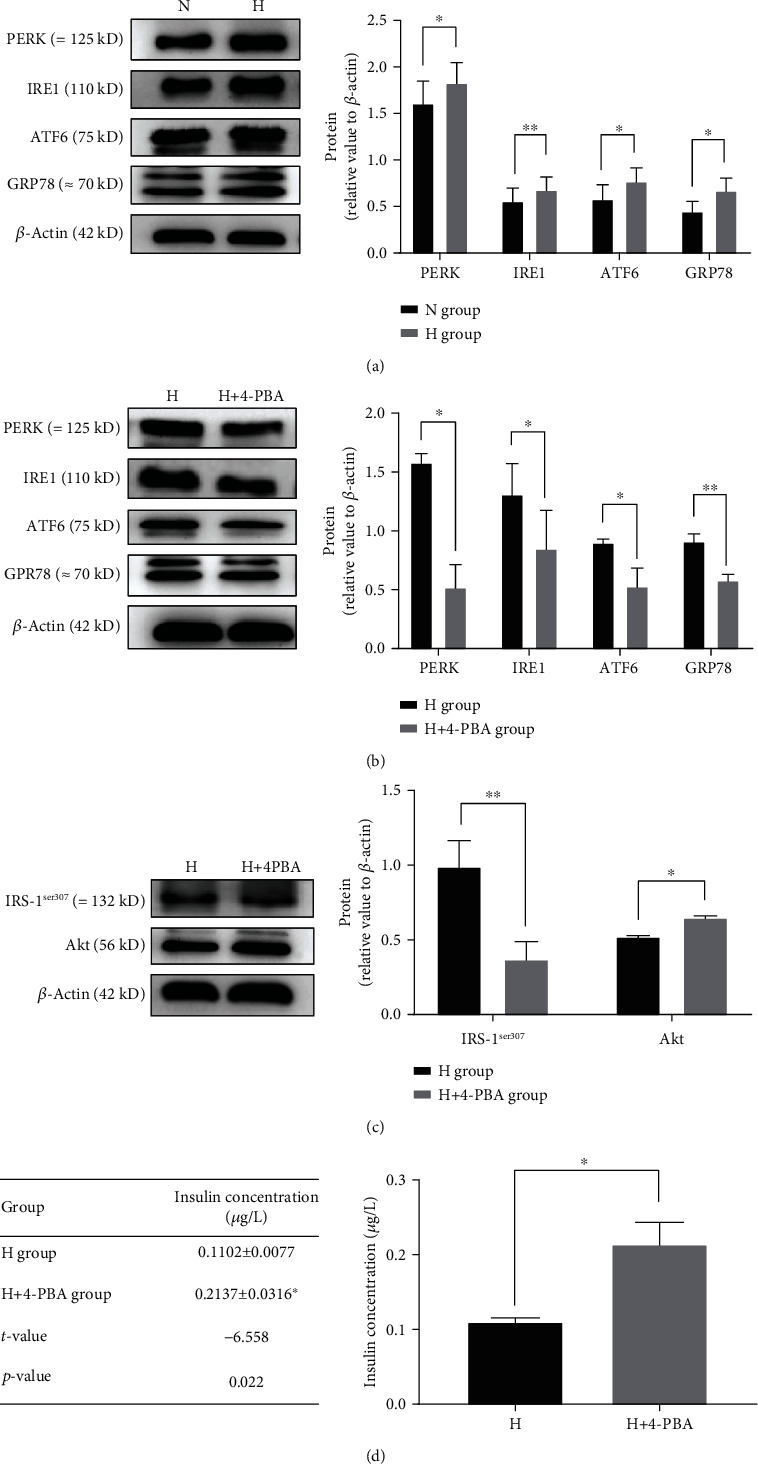
Expression of specific proteins of ER and insulin in *β*-cells treated with different concentrations of T3. N group: normal-concentration T3 (5 pg/mL); H-group: high-concentration T3 (20 pg/mL); H+4-PBA group: high-concentration T3 (20 pg/mL)+4-PBA (1 mmol/L). (a) Western blotting analysis showed that the expression of GRP78, PERK, IRE1, and ATF6 in the H group was significantly higher than that in the N group. (b) The expression of GRP78, PERK, IRE1, and ATF6 in the H+4-PBA group was significantly lower than that in the H group. (c) The expression of p-IRS-1^ser307^ in the H+4-PBA was significantly lower than that in the H group, whereas Akt expression was higher than that in the H group. (d) ELISA showed that the concentration of insulin in the supernatant of the H group was significantly lower than that of the H+4-PBA group (*n* = 3, ^∗^*p* < 0.05, ^∗∗^*p* < 0.01). ELISA: enzyme-linked immunosorbent assay.

We treated MIN6 cells with T3 and 4-PBA, respectively, and collected the proteins of these cells. Western blotting analysis showed that compared with the normal-concentration T3 group, the high-concentration T3 group exhibited an increase in the expression of GRP78, PERK, IRE1, and ATF6, which are specific proteins of the ERS (Figure 3(a)). As mentioned above, high concentrations of T3 upregulated IRS-1^ser307^, downregulated Akt, and decreased the insulin secretion of islet *β*-cells. These processes were all reversed by 4-PBA, an inhibitor of ERS (Figures 3(b)–3(d)).

### 3.4. High T3 Induces ERS-Associated Apoptosis in Pancreatic Cells

**Figure 4:**
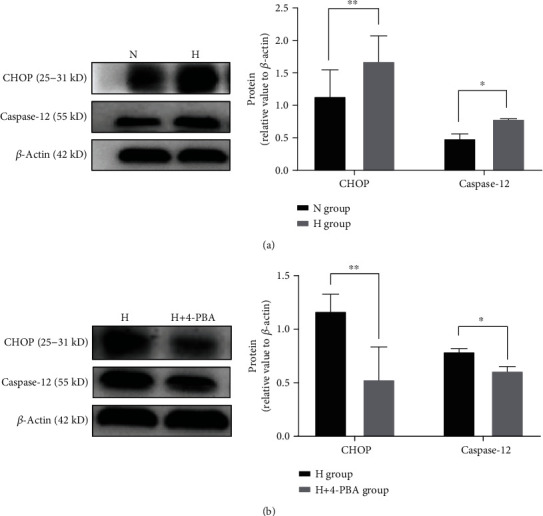
Expression of CHOP and caspase-12 in *β*-cells treated with different concentrations of T3. Western blotting analysis showed that (a) the expression of CHOP and caspase-12 in the H group was significantly higher than that in the N group, and (b) the expression of CHOP and caspase-12 in the H+4-PBA group was significantly lower than that in the H group (*n* = 3, ^∗^*p* < 0.05, ^∗∗^*p* < 0.01).

Western blotting analysis showed that compared with the normal-concentration T3 group, the high-concentration T3 group exhibited increased expression of CHOP and caspase-12, which are special apoptosis-related proteins of the ERS (Figure 4(a)). These processes were reversed by 4-PBA (Figure 4(b)).

## 4. Discussion

Studies have shown that glucose metabolic disorders and insulin resistance are common in patients with hyperthyroidism [15]. High levels of T3 can cause peripheral insulin resistance in the liver and adipose tissues [16]. However, studies on insulin resistance in cells induced by high T3 levels are limited. *β*-cells secreting insulin are regulated by insulin signal transduction. Insulin resistance in *β*-cells can result in a decrease in insulin secretion [3]. Insulin receptor substrate (IRS-1) is an important target of the insulin signal transduction pathway in *β*-cells and plays an important role in the development of insulin resistance. Insulin resistance is not the only factor. There are many factors leading to *β*-cell death. But previous studies have shown that insulin resistance can cause severe apoptosis [17, 18]. And this study only focused on insulin resistance.

Activation of IRS-1 wild-type *β*-cells could induce insulin secretion in *β*-cells, but insulin secretion could not be detected in IRS-1-/-*β*-cells [19–21]. Currently, serine phosphorylation at the ser307 site of IRS-1 has been extensively studied. When serine phosphorylation at the ser307 site of IRS-1 is activated, tyrosine phosphorylation is inhibited and blocks the insulin signal transduction pathway. Therefore, IRS-1^ser307^ increased, but insulin secretion of pancreatic *β*-cells decreased. [22]. Akt, also known as PKB, is also an important target in the insulin signal transduction pathway [23]. Activated Akt can phosphorylate many substrates and promote insulin secretion. When insulin resistance occurs, Akt expression is downregulated. Our study found that the expression of p-IRS-1^ser307^ in the high-level T3 group increased, while the expression of Akt decreased. The fact that sodium vanadate, an inhibitor of phosphorylation of IRS-1^ser307^, can recover insulin secretion indicates that high levels of T3 can inhibit insulin signal transduction in *β*-cells via the IRS-1/PI3-K/Akt pathway and result in insulin resistance [14, 24]. To reveal whether high levels of T3 lead to ERS, we detected ERS marker proteins and ERS apoptosis marker proteins. We found that high levels of T3 significantly increase the expression of ERS characteristic proteins PERK, IRE1, ATF6, and GRP78 as well as ERS apoptosis marker proteins CHOP and caspase-12 and could be corrected by the ERS inhibitor 4-PBA. The results show that high levels of T3 could induce *β*-cell ERS and apoptosis.

Our study was mainly carried out in MIN6 cell line. Previous studies showed that insulin secretion of *β*-cell line MIN6 increased significantly at high glucose concentration, and its insulin secretion and S-type response curve were similar to those of normal islet cells. Further studies on insulin secretion, glucose transport, glycooxidative phosphorylation, and glucose utilization of insulin *β*-cell line MIN6 showed that the cell components in MIN6 cells that regulate glucose concentration-dependent insulin secretion are similar to normal islets in quality and quantity [25, 26].

During ERS, the PERK-eIF2 *α* pathway is activated, and the expression of ATF4 is increased, which leads to the upregulation of TRB3 expression. As a negative feedback regulator of the insulin signal transduction pathway, TRB3 can affect the insulin signal transduction pathway by inhibiting the function of Akt, which then causes insulin resistance [27]. PKR can be activated by ERS, which not only directly inhibits tyrosine phosphorylation of IRS-1^ser307^ and causes insulin resistance but also reduces the phosphorylation of Akt by activating JNK and inhibits insulin signal transduction, indirectly leading to insulin resistance. In this study, after the application of ERS inhibitor 4-PBA, ERS was relieved, whereas IRS-1^ser307^ was significantly downregulated, the expression of Akt was significantly upregulated, and insulin secretion also increased. These findings suggest that high levels of T3 can induce insulin resistance and decrease *β*-cell insulin secretion via ERS.

When ERS is too strong, the apoptosis signal pathway is initiated. This is an independent apoptotic pathway. CHOP and caspase-12 are specific apoptotic proteins that are related to ERS [12, 28, 29]. High levels of T3 lead to the significant upregulation of CHOP and caspase-12, which further indicates that high levels of T3 can induce *β*-cell apoptosis through ERS and result in a decrease in insulin secretion in *β*-cells. This may also be an indirect mechanism for insulin resistance in *β*-cells.

In sum, our study suggests that high levels of T3 can induce ERS and result in insulin resistance and insulin secretion in *β*-cells, thereby contributing to the pathogenesis of diabetes.

## Data Availability

The data used to support the findings of this study are available from the corresponding authors upon request.
